# From the (Epi)Genome to Metabolism and Vice Versa; Examples from Hematologic Malignancy

**DOI:** 10.3390/ijms22126321

**Published:** 2021-06-12

**Authors:** Panagiota Karagianni, Stavroula Giannouli, Michael Voulgarelis

**Affiliations:** 1 Department of Pathophysiology, School of Medicine, National and Kapodistrian University of Athens, 11527 Athens, Greece; pkaragia@med.uoa.gr; 2 Hematology Unit, Second Department of Internal Medicine, School of Medicine, National and Kapodistrian University of Athens, 11527 Athens, Greece; sgiannoul@med.uoa.gr

**Keywords:** hematologic malignancy, leukemia, lymphoma, myeloma, myelodysplastic syndrome, genetic, epigenetic, metabolic

## Abstract

Hematologic malignancies comprise a heterogeneous group of neoplasms arising from hematopoietic cells or their precursors and most commonly presenting as leukemias, lymphomas, and myelomas. Genetic analyses have uncovered recurrent mutations which initiate or accumulate in the course of malignant transformation, as they provide selective growth advantage to the cell. These include mutations in genes encoding transcription factors and epigenetic regulators of metabolic genes, as well as genes encoding key metabolic enzymes. The resulting alterations contribute to the extensive metabolic reprogramming characterizing the transformed cell, supporting its increased biosynthetic needs and allowing it to withstand the metabolic stress that arises as a consequence of increased metabolic rates and changes in its microenvironment. Interestingly, this cross-talk is bidirectional, as metabolites also signal back to the nucleus and, via their widespread effects on modulating epigenetic modifications, shape the chromatin landscape and the transcriptional programs of the cell. In this article, we provide an overview of the main metabolic changes and relevant genetic alterations that characterize malignant hematopoiesis and discuss how, in turn, metabolites regulate epigenetic events during this process. The aim is to illustrate the intricate interrelationship between the genome (and epigenome) and metabolism and its relevance to hematologic malignancy.

## 1. Introduction

Hematologic malignancies are myeloid and lymphoid neoplasms arising as a consequence of the disruption of normal hematopoiesis. The group comprises a diverse set of diseases, including chronic and acute lymphocytic and myeloid leukemias, B-, T-, and NK-cell lymphomas, plasmacytic myelomas, as well as myeloproliferative neoplasms and myelodysplastic syndromes (MDS) [1]. Besides their commonalities, the above conditions also exhibit marked differences in terms of genetic etiology, clinical course and prognosis, which also guide diverse therapeutic approaches.

In comparison to solid tumors, in hematologic malignancies neoplastic cells are generally more easily accessible, and can often be isolated from the patient’s peripheral blood. This has provided basic research with a valuable resource, which has allowed for deeper understanding of the underlying biology and has established the basis of development of effective treatments. Genetic analysis has identified a growing list of chromosomal abnormalities and point mutations that are recurrent in various types of blood neoplasms. Some of them are considered driver mutations, as they are critically involved in the neoplastic transformation [2], while others accumulate in the process, as they provide selective advantage to the rapidly proliferating tumor cell. In addition to genetic alterations, epigenetic changes, i.e., modifications of chromatin which do not affect the DNA sequence, are also key components of the initiation and evolution of hematologic malignancies, primarily via their effect on regulating the expression of numerous genes implicated in cellular processes such as proliferation and differentiation [3]. Both genetic and epigenetic events also contribute to the metabolic rewiring of the cancer cell, which supports its increased energetic and biosynthetic demands in a dynamic microenvironment. Interestingly, signaling between the nucleus and metabolism is bidirectional, as metabolites also regulate epigenetic modifications via direct and indirect mechanisms.

In this article, we provide an overview of the metabolic reprogramming characterizing hematopoietic cells following malignant transformation and the main genetic alterations that drive this reprogramming. Furthermore, we discuss how metabolic changes signal back to the cell’s nucleus, shaping the epigenetic landscape and in this way affecting transcription. Although the specific events in place vary widely across the spectrum of tumor cell states, the aim is, through selected examples, to illustrate the intricate interrelationship between the genome (and epigenome) and metabolism and its relevance to hematologic malignancy.

## 2. Normal Hematopoiesis and Metabolic Regulation

**Figure 1:**
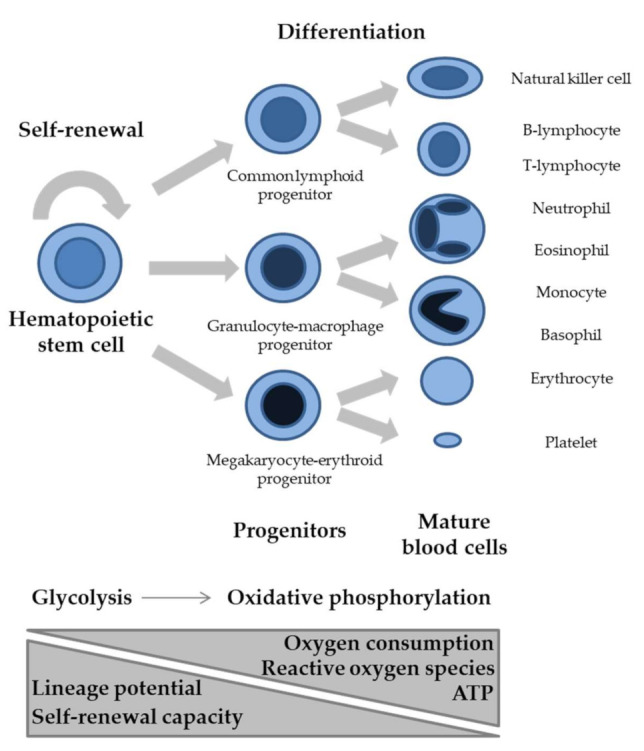
Simplified diagram of hematopoiesis, with selected cells provided as examples. ATP: adenosine triphosphate.

Hematopoiesis is a dynamic developmental process through which a small pool of stem cells provides lifelong supply of all mature blood cell types of the organism, based on a series of tightly regulated and hierarchically organized proliferation and differentiation events (Figure 1). In humans, hematopoiesis starts in the yolk sac, and then transitions first to the liver and finally to the bone marrow and thymus. Hematopoietic stem cells occupy the top of the hematopoietic hierarchy and combine the capacity for self-renewal and the potential to differentiate to virtually any blood cell type, two properties that are fundamental for tissue homeostasis. Depending on the organismal needs, hematopoietic stem cells can exist in a quiescent state for long periods of time before entering active proliferation in response to stimulation. Proliferation can give rise to new stem cells ensuring the maintenance of an adequate pool, as well as progenitors that will differentiate towards mature cell types. In the process of differentiation, cells generally become gradually more committed to specific lineages and at the same time limited in terms of the repertoire of cell types they can produce.

The fine balance between the above cellular states relies on complex interactions involving factors from the cellular microenvironment, signaling pathways, and transcriptional and epigenetic regulatory events, and is closely linked with metabolic adaptations. Specific examples are provided in the text to follow. Metabolic adaptations ensure that the cell tailors its nutrient supplies in the optimal way to meet its energetic and biosynthetic demands. In a simplified view, a proliferating cell will generally use its available energy and metabolites in anabolic reactions to synthesize biomolecules that will serve as building blocks required for cell division, while a differentiated cell will rely primarily on reactions that produce energy to maintain its organization and perform its cellular functions. A determining factor in metabolic processes is oxygen availability. In the context of hematopoiesis, oxygen availability changes dramatically depending on the cell’s microenvironment, as the bone marrow, where hematopoietic stem cells reside, is hypoxic, while most differentiated cells live under normoxic conditions.

**Figure 2:**
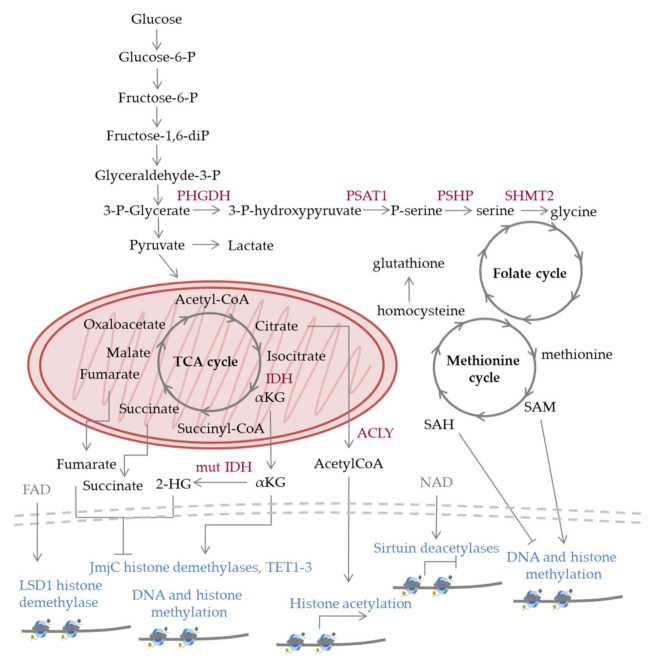
Schematic representation of selected metabolic pathways and enzymes discussed in the text. The reactions occur in normal cells; however, mutations (e.g., in IDH (mut IDH)) or aberrant regulation observed in malignant cells can lead to shift towards specific pathways versus others and altered levels of metabolites compared to normal cells, which has an impact on epigenetic processes. Nucleosomes are depicted at the lower part of the figure; epigenetic modifications can lead to gene activation (arrows), repression (flat-ended lines), while in many cases their effect varies depending on the genomic location, cell type, and other factors. 2-HG: 2-hydroxyglutarate, α-KG: α-ketoglutarate, acetyl-CoA: acetyl coenzyme A, FAD: flavin adenine dinucleotide, IDH: isocitrate dehydrogenase, LSD1: lysine specific demethylase 1, NAD: nicotinamide adenine dinucleotide, PHGDH: phosphoglycerate dehydrogenase, PSAT1: phosphoserine aminotransferase 1, PSHP: phosphoserine phosphatase, SAH: S-adenosylhomocysteine, SAM: S-adenosylmethionine, SHMT2: serine hydroxymethyltransferase-2, TET1-3: ten-eleven-translocation 1-3.

In terms of preferred metabolic pathways, the general view is that undifferentiated hematopoietic stem cells, which have limited oxygen availability in the bone marrow, undertake primarily glycolytic reactions, while as cells differentiate, they gradually shift towards oxidative phosphorylation. Glycolysis is a sequence of reactions taking place in the cytoplasm, through which one molecule of glucose is converted into two molecules of pyruvate (Figure 2). This process does not require molecular oxygen, and for each glucose molecule broken down, there is a net gain of two molecules of adenosine triphosphate (ATP) and two molecules of nicotinamide adenine dinucleotide (NAD^+^), reduced (NADH). Under anaerobic conditions, cells convert pyruvate into lactate, with NADH giving up its electrons to generate NAD+, required for glycolysis to continue [4]. Glycolysis is energetically less efficient than oxidative phosphorylation, described below, but provides proliferating cells anabolic intermediates serving as building blocks to support cell division. On the other hand, in most normal differentiated cells, which have left the bone marrow and live under aerobic conditions, pyruvate is rapidly transported into the mitochondria, where it is converted to acetyl-coenzyme A (acetyl-CoA). The latter will then enter the citric acid cycle, also taking place in the mitochondria, leading to the production of two molecules of CO_2_, three molecules of NADH, one molecule of GTP, and one molecule of flavin adenine dinucleotide reduced (FADH_2_) in each turn, as well as carbon-containing intermediates, such as oxaloacetate and α-ketoglutarate. Via oxidative phosphorylation, which requires molecular oxygen, the electrons of NADH and FADH_2_ enter the electron transport chain, embedded in the mitochondria inner membrane, and produce ATP. Through this process, the net energy gain from a single glucose molecule is 30–36 molecules of ATP, which is 15–18 times higher than that produced through glycolysis.

The shift towards oxidative phosphorylation during the differentiation of hematopoietic progenitors is accompanied by increased mitochondrial biogenesis and activity as well as increased production of reactive oxygen species (ROS). ROS derive from the transfer of free electrons to molecular oxygen and can cause damage to DNA, lipids and proteins. Thus, the preference of hematopoietic stem cells towards glycolysis versus oxidative phosphorylation also offers the advantage of limiting their exposure to ROS, which could compromise stemness or introduce mutations with catastrophic consequences for hematopoiesis. Besides their damaging effects, within certain levels, ROS can exert signaling roles. In the form of H_2_O_2_, they can exert direct and indirect regulation of transcriptional programs implicated in hematopoiesis [5]. It should also be noted that the effects of H_2_O_2_ extend beyond its cell-autonomous functions, as, via its relatively long half-life and its ability to pass through the cell membrane, the molecule can also mediate paracrine signaling.

The effects of metabolites and factors of the microenvironment, such as oxygen availability and signaling molecules, on hematopoiesis and the associated metabolic changes are mediated by a highly organized network of transcription factors and epigenetic modifiers, which activate or repress the expression of specific gene programs. For instance, central regulators of the response to hypoxia are the hypoxia inducible factors (HIFs) [6], which activate the transcription of glycolytic enzymes and other genes that favor anaerobic metabolism rather than oxidative phosphorylation. HIFs also reduce ROS production and are critical for the maintenance of stem cell quiescence [7]. Chromatin modifying enzymes generate a landscape that poises specific gene sets for activation or repression, determined by the spatially and temporally regulated activity of transcription factors. Tight regulation of gene expression programs will ultimately determine differentiation as well as the adaptation of the cell to its microenvironment.

## 3. Metabolic Changes in Hematologic Malignancies

Cancer cells exhibit profound metabolic differences from normal cells, resulting from the combined action of deregulated intracellular signaling pathways, as well as signals coming from their microenvironment [8]. One of the first metabolic differences between tumor and normal cells to be appreciated was their shift from oxidative phosphorylation towards glycolysis even under conditions of oxygen availability, producing large amounts of lactate, known as the Warburg effect [9]. Glycolysis allows cancer cells to use available nutrients for the synthesis of macromolecules to support their rapid growth and proliferation [10] and has been described in several solid and hematologic cancers, including acute myeloid leukemia (AML) and acute lymphoblastic leukemia (ALL) cells [11]. In leukemia initiating cells (LIC), which comprise a rare subpopulation of leukemic cells with stem cell properties, a high glycolytic rate has been proposed to determine homing and cell division [12]. However, the dependence on glycolysis is not universal across all types of cancer cells. By monitoring the cytosolic NADH/NAD+ ratio in a transgenic mouse model engineered to express a highly responsive fluorescent NADH/NAD+ sensor as well as in primary human B cell acute lymphoblastic leukemia (B-ALL) cells, increased rates of oxidative phosphorylation were detected, presumably maintained by the function of the pyruvate dehydrogenase complex component X (PDHX), which facilitates the entry of pyruvate into the TCA cycle [13]. Moreover, the increased oxidative phosphorylation rates of B-ALL cells were associated with higher leukemogenic activity and their preference to reside in the vascular niche in the bone marrow [13]. Although the role of the tumor microenvironment is important, the factors that determine the energetic preferences of different cell types remain largely unknown.

Increased ROS production has been detected in several types of hematologic cancer cells and can result from oxidative phosphorylation or other mechanisms, including constitutive activation of the NADPH oxidase 2 [14,15]. Increased ROS levels are observed in the majority of AML patients [16]. However, cancer cells alter their redox homeostasis in ways that allow them to withstand the effects of increased ROS production. In AML cells, ROS have been shown to promote glucose uptake and regulate several aspects of carbohydrate and sphingolipid metabolism [17]. Redox status is also affected by glutathione, which is the main antioxidant defense used by the cell against oxidative stress, as well as by the levels of iron. Iron can have oxidative or reductive functions in various biochemical reactions. In multiple myeloma (MM), increased iron uptake and intracellular levels have been demonstrated to promote cell growth. Experiments in myeloma cell lines have shown that the increased cellular iron content induces ROS increase, which in turn stimulates an adaptive response mediated by the activation of the redox regulating genes and mitochondrial biogenesis and fitness [18]. The above mechanisms have also been proposed to be of relevance in the development of treatment resistance.

The observation that specific metabolites were found to be upregulated in various tumors led to their characterization as oncometabolites. Examples relevant to several hematopoietic malignancies include the TCA intermediates fumarate, succinate, icocitrate and α-ketoglutarate (α-KG). The latter is produced by oxidative decarboxylation of isocitrate by isocitrate dehydrogenase (IDH) enzymes and oxidative deamination of glutamate by glutamate dehydrogenase. Another oncometabolite is 2-hydroxyglutarate (2-HG), a byproduct of reductive glutamine carboxylation, which drives malignant transformation and tumor progression when overproduced, via multiple pathways [19]. For instance, 2-HG inhibits complex IV and ATP synthase and disrupts the respiratory chain activity in the mitochondria [20]. In addition, 2-HG modulates the tumor microenvironment through its non-cell-autonomous impact on the immune system [21]. It should be noted that the oncogenic effects of 2-HG largely depend on the specific cell type. Mechanistic insight into the deregulation of the aforementioned oncometabolites, as well as their effects in the context of the transformed hematopoietic cell will be provided below.

Another metabolic pathway upregulated in several hematologic malignancies, including Burkitt’s lymphoma (BL), is the one-carbon (1C) metabolism pathway [22]. This pathway integrates the folate cycle, the methionine cycle and the trans-sulfuration pathway and provides 1C units (methyl groups) for various processes, such as de novo synthesis of nucleotides, amino acids, phospholipids and other molecules and for the regeneration of mitochondrial NADH, NADPH, and ATP [23]. A major source of one carbon to fuel the pathway is serine, which can be synthesized from the glycolytic intermediate 3-phosphoglycerate in three steps. In the first step, 3-phosphoglycerate is converted into 3-phosphohydroxypyruvate by phosphoglycerate dehydrogenase (PHGDH). In the second step, 3-phosphohydroxypyruvate is converted to phosphoserine by phosphoserine aminotransferase 1 (PSAT1), and in the last step, phosphoserine is converted into serine by phosphoserine phosphatase (PSPH). Through the action of the mitochondrial enzyme serine hydroxymethyltransferase-2 (SHMT2), serine is converted to glycine, generating a 1C unit that can be used to produce NADPH and S-adenosyl methionine (SAM) through the folate and methionine cycles [23,24,25,26]. SAM serves as the major methyl donor in several reactions, including the methylation of histones and DNA [27]. The methionine cycle is also interconnected with redox homeostasis, as it provides cysteine for the synthesis of glutathione.

Similarly, the mevalonate pathway, which produces isoprenoid precursor units, is also commonly upregulated in cancer cells, particularly those carrying *TP53* mutations [28]. This pathway provides metabolites used for the synthesis of cholesterol as well as for several post-translational modifications, such as protein prenylation, a modification mediating membrane attachment and protein–protein interactions in inter- and intra-cellular signaling pathways.

Among tumor cells there is significant heterogeneity with respect to metabolic alterations. In fact, the metabolic profile can be highly mutable even within the same tumor during the course of the disease. However, it appears that certain profiles are associated with specific cell types [29,30], presumably due to genetic and epigenetic characteristics of their lineage as well as the effect of their specific microenvironment. Regardless of its mechanistic explanation, metabolic profiling is becoming increasingly appreciated with the development of novel analytic technologies. In an unbiased metabolomics analysis of differentiating myeloid lineage cells from MDS bone marrow aspirates, a profile coordinated primarily by the Warburg effect was observed, characterized by the accumulation of glycolytic intermediates, decreased pyruvate/lactate and NAD/NADH ratios, and increased ADP/ATP ratio. Moreover, MDS cells also displayed increased levels of 1C-derived SAM and 2-HG compared to control cells. Interestingly, respiratory capacity and redox homeostasis were proposed to determine the ability of cells to compensate for the ongoing metabolic stress, which eventually functions as a driving force for the selection of blasts with increased oncogenic potential and shapes the MDS clinical phenotype [31].

## 4. Mutations in Metabolic Enzymes and Their Transcriptional Regulators

Direct evidence on the contribution of metabolic programs to the malignant phenotype comes from genetic data supporting that mutations in metabolic genes are key drivers in hematologic malignancies. Such examples are mutations in the genes encoding isocitrate dehydrogenase 1 (IDH1) and 2 (IDH2). IDH normally catalyzes the conversion of isocitrate to α-KG, with IDH1 localized in the cytosol and peroxisomes and IDH2 in the mitochondria. IDH mutations are a recurrent theme in several hematologic malignancies, including AML and angioimmunoblastic T cell lymphoma (AITL). IDH1 mutations are detected in ~5–10% of patients with AML and IDH2 in 15–20% of patients with AML and are generally more frequent in patients with normal karyotype [2,32]. Examples include neomorphic missense mutations on R132 of IDH1 as well as R140 and R172 of IDH2, which alter the catalytic properties of the enzymes towards the conversion of α-KG to (2R)-hydroxyglutarate (2R-HG) [32,33]. Such mutations can cause extensive metabolic reprogramming, with disruption of glucose, lipid and phosphocholine metabolism, and increased ROS production [34,35,36,37]. Mutant cells often display a shift in metabolite flux through the TCA cycle and increased consumption of NADPH and α-KG, increased levels of glycine, serine, and threonine, and depletion of aspartate, glutamate and *N*-acetylated amino acids [37]. The effects of 2R-HG accumulation are widespread and its action on specific enzymes will be discussed below. The above changes have the potential of disrupting the balance between differentiation and cellular proliferation, thus promoting tumor initiation and progression [38,39]. However, it is noteworthy that the phenotypic outcome of IDH mutations can vary depending on the cell type, as the same mutation can cause a different effect in myeloid versus lymphoid cells [40]. There are multiple other examples of direct connection between the activity of metabolic enzymes and tumorigenesis. For instance, deficiencies of the TCA enzymes fumarate hydratase and succinate dehydrogenase have been detected in several tumors, characterized by the accumulation of fumarate and succinate, or succinate, respectively [41], while genetic variants of drug metabolizing enzymes, such as the P450 cytochrome (CYP), glutathione S-transferase theta 1 and mu 1 (GSTT1 and GSTM1), and NAD(P)H menadione oxidoreductase 1, have been associated with increased risk of developing MDS [42]. Hematologic malignancies also often carry genetic alterations on genes encoding key regulators of metabolic gene expression. For example, chromosomal translocations t(8;14), t(8;22), or t(2;8), which juxtapose *MYC* to the heavy- or light-chain immunoglobulin gene regulatory regions, have been described both in B-ALL and in non-Hodgkin lymphomas (NHL), mainly BL, and diffuse large B-cell lymphomas (DLBCL) [43,44]. These rearrangements lead to the overexpression of the *MYC* gene, which encodes for a leucine zipper transcription factor regulating various genes involved in cellular proliferation and differentiation. In addition, MYC regulates several metabolic genes, implicated in glycolysis, glutaminolysis, nucleotide and lipid synthesis and others [45]. For instance, the genes encoding for the PHGDH and PSAT1 enzymes of the serine biosynthetic pathway are MYC transcriptional targets. Overall, changes in MYC expression as a consequence of genetic alterations confer cancer cells a metabolic flexibility to allow them to grow and proliferate in unfavorable conditions [46].

**Figure 3:**
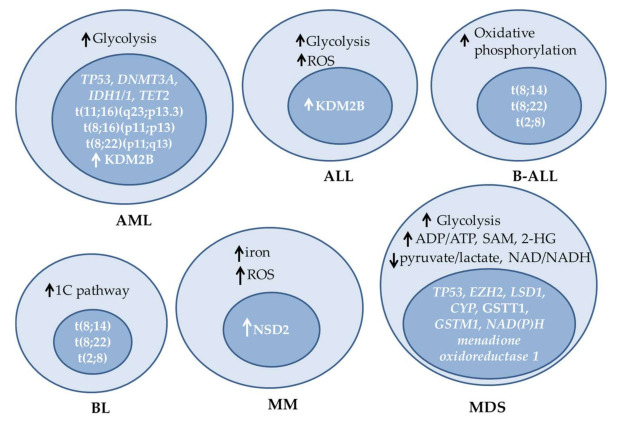
Examples of metabolic alterations, mutations and changes in expression of epigenetic enzymes observed in different hematologic malignancies. ALL: acute lymphoblastic leukemia, AML: acute myeloid leukemia, B-ALL: B-cell ALL, BL: Burkitt’s lymphoma, MDS: myelodysplastic syndromes, MM: multiple myeloma, ROS: reactive oxygen species, SAM: S-adenosylmethionine.

The *TP53* gene, encoding for a master regulator of many cellular processes, is also frequently mutated in hematologic malignancies (Figure 3). Germline *TP53* mutations in individuals affected by Li–Fraumeni syndrome have been linked with a predisposition to cancer, including leukemia [47], while somatic *TP53* mutations in AML are generally associated with acute disease, inferior response to therapy and poor prognosis [48]. Both gain- and loss-of-function mutations have been described, and a significant proportion of them affect the DNA binding domain of the protein. In MDS, genetic profiling of a large cohort recently uncovered a critical effect of *TP53* allelic state on the disease phenotype, with patients harboring biallelic mutations being at increased risk of death and leukemic transformation, and showing poor response to therapy, and patients with monoallelic mutations not differing significantly from patients with wild-type *TP53* in the aforementioned parameters [49]. Although the role of TP53 in cancer has been primarily viewed through its effect on regulating DNA repair, cell cycle and apoptosis, its metabolic functions are also relevant in hematologic malignancy. Via transcriptional and other mechanisms, TP53 modulates glycolysis, oxidative phosphorylation, as well as ROS levels [50]. Metabolic transcriptional targets of TP53 include the genes encoding phosphate-activated mitochondrial glutaminase (*GLS2)*, a key enzyme in the conversion of glutamine to glutamate, and regulator of glutathione (GSH) synthesis and energy production [51], and cytochrome *c* oxidase (*SCO2*) [52]. TP53 is also a phosphorylation substrate for the AMP-activated protein kinase (AMPK) [53], which is the main metabolic sensor of the cell, activated upon the depletion of intracellular ATP, under nutrient deprivation, hypoxia, and mitochondrial stress. In cultured AML cells, upon metabolic stress induced by the pyruvate dehydrogenase kinase-1 (PDK1) inhibitor dichloroacetate (DCA), TP53 is activated in an AMPK-dependent manner and regulates the transcription of its metabolic targets, while it also causes cell cycle arrest [54]. However, the mechanisms regulating the stability of TP53 depend on its mutational status, with specific mutants shown to inhibit autophagic pathways [55].

Besides transcription factors, epigenetic regulators are also commonly mutated in hematologic malignancies. Epigenetic regulators can be broadly categorized into enzymes that add or remove covalent marks on histones or the DNA, chromatin remodelers and factors that recognize and bind to specific chromatin marks, serving as scaffolds for the recruitment of complexes that mediate chromatin-templated events. Some epigenetic factors have global functions, while others regulate more limited gene sets, which also depend on the cell type and state. Therefore, mutations on the genes encoding them can have widespread or more specific effects. In the context of this review, we focus on factors directly or indirectly affecting metabolic processes. One such example is the enhancer of zeste homolog 2 (EZH2), the catalytic subunit of the Polycomb Repressive Complex 2 (PRC2), which is a histone H3 lysine 27 (H3K27me2/3) methyltransferase [56]. A variety of both gain- and loss-of-function mutations of *EZH2* have been described in several hematologic malignancies, supporting that tight regulation of its activity is critical for cellular homeostasis across different cell types. For instance, *EZH2* mutations occur in follicular lymphoma (FL) [57,58], germinal center B-cell like (GCB) DLBCLs [59,60,61,62,63], MDS [64], myeloproliferative neoplasms (MPN) [65], and juvenile myelomonocytic leukemia (JMML) [66,67]. A key EZH2 residue found mutated in hematologic malignancies is tyrosine 641 (Y641), located in the catalytic SET domain of the protein. In contrast to the wild-type enzyme, which preferentially methylates unmethylated and monomethylated H3K27, biochemical studies have demonstrated that several Y641 substitutions result in increased activity towards dimethylated H3K27 (H3K27me2) [68,69]. Consequently, in the heterozygous state, such mutations result in a global increase in H3K27me3, and a decrease in H3K27me1 and H3K27me2 levels [70,71]. Since H3K27me3 generally functions as a repressive mark, their presence is accompanied by transcriptional repression of many genes with direct or indirect implication in the malignant phenotype, including genes with metabolic functions. In MM, increased levels of H3K27me3 correlate with advanced stages of the disease [72]. It should be noted that the deregulation of EZH2 in neoplasms can also arise as a consequence of other mechanisms, besides *EZH2* gene mutations, such as transcriptional overexpression, changes in splicing, e.g., due to serine/arginine-rich splicing factor 2 (SRSF2) mutations, and changes in its chromatin distribution [73]. The latter have been observed in cells carrying mutations of the PRC2 associated protein additional sex combs-like 1 (ASXL1) [74].

The redistribution of EZH2 has also been observed in cells with high multiple myeloma SET domain (MMSET) levels. *MMSET* encodes for the histone methyltransferase NSD2, which mainly catalyzes histone H3 dimethylation (H3K36me2). Its overexpression is observed in approximately 15% of MM patients as a consequence of the translocation t(4;14)(p16;q32) and is considered to play a key role in the neoplastic transformation [75]. Interestingly, the global increase in histone H3 lysine 36 methylation (H3K36) observed in cells overexpressing NSD2 was also accompanied by a decrease in H3K27 methylation [76]. However, despite the global reduction in H3K27 methylation, specific loci display increased levels of this modification, due to the redistribution of EZH2 across the genome [77]. The above observations led to the idea of an antagonistic effect between H3K36 and H3K27 methylation. Kinetic measurements further revealed that in cells with *MMSET* overexpression, the effective rate constants of H3K36 mono- and dimethylation are increased. Moreover, in these cells, H3K27 monomethylation constants are also increased, but the effective rate constants for its reversal are even higher, thus explaining the global reduction in H3K27 methylation. However, despite this global reduction, due to the redistribution of EZH2 across the genome, specific loci display increased H3K27 methylation [78]. Last, it is worth noting that aberrant activity of KDM2B, which catalyzes the demethylation of H3K36me2, has also been observed in hematologic malignancies, further illustrating the critical significance of tight regulation of this modification for hematopoietic cell homeostasis. In fact, both upregulation and downregulation of the expression of KDM2B have been observed in different hematologic malignancies and its role can differ depending on the specific cell type. The *KDM2B* gene was identified as a common integration site in Moloney murine leukemia virus-induced T cell lymphomas, while its elevated expression has also been observed in patients with AML or ALL. Moreover, differential methylation of the *KDM2B* gene has been described in cell lines derived from Epstein–Barr virus (EBV)-associated endemic BL compared to cells derived from EBV-negative sporadic BL. In mice, *KDM2B* overexpression was also shown to induce mitochondrial metabolic activation [79].

A common rearrangement in leukemia involves the chromosomal region containing the gene encoding for KMT2A. KMT2A has histone methyltransferase activity, with specificity for H3K4, and is ubiquitously expressed in hematopoietic cells. It has multiple functional domains, some of them mediating interaction with other transcriptional regulators, such as the CREB-binding protein (CBP) [80,81], and regulates the transcription of a number of genes, via direct or indirect mechanisms. One of its direct targets is the gene encoding for 6-phosphofructo-2-kinase/fructose-2,6-biphosphatase 4 (PFKFB4), an enzyme converting fructose 6-biphosphate into fructose 2,6-bisphosphate. Increased levels of PFKFB4 have been proposed to promote acute monocytic leukemia (AMoL) progression by stimulating glycolytic flux to the TCA cycle and lactate, and are generally associated with poor prognosis [82].

## 5. Metabolic Effects on Epigenetics

The interplay between the genome and the metabolome is bidirectional, as metabolic changes can affect gene expression via epigenetic mechanisms. Importantly, both acetylation and methylation, which comprise the main transcriptional regulating epigenetic modifications, are affected by the availability of the acetyl donor acetyl-CoA and the methyl donor SAM, respectively, both of which can be altered as a consequence of deregulated metabolic pathways. Other metabolites are also being used as cofactors by specific histone and DNA modifying enzymes, thereby their intracellular levels are tightly interrelated with the end products of these enzymatic activities [83].

The significance of accurate regulation of histone acetylation for the homeostasis of hematopoietic cells is underscored by the fact that genomic rearrangements leading to the production of histone acetyltransferase (HAT) fusion proteins are found in multiple rare AML such as t(11;16)(q23;p13.3), t(8;16)(p11;p13), and t(8;22)(p11;q13) [84]. The acetylation of histones is a key component of transcriptional regulation and is generally associated with active genes. Thus, the above rearrangements lead to hyperacetylation and aberrant activation of the targeted promoters. It should also be noted that HATs can also catalyze the acetylation of non-histone substrates, which could also contribute to the observed phenotypes, while in some cases their effect on promoting AML is mediated by their scaffolding functions rather than their enzymatic activities. It is worth mentioning that several metabolic enzymes are also regulated by acetylation [85]. The acetyl-CoA pool is derived from citrate, via the enzymatic action of ATP citrate lyase (ACLY) [86]. ACLY is also activated by the PI3K/Akt/mTOR axis to maintain acetyl-CoA in the nucleus under conditions of starvation [87]. The levels of acetyl-CoA and histone acetylation are also affected by the activity of AMPK. In cultured AML cells, AMPK deletion led to reduced acetyl-CoA and histone acetylation levels [88]. However, the effect of AMPK on epigenetic regulation extends beyond acetylation, as the enzyme also phosphorylates H2B [89], H3 [90], as well as several histone and DNA modifying enzymes, including EZH2 [91], DNA methyltransferase 1 (DNMT1) [92], and TET2 [93,94]. Another link between metabolism and epigenetic regulation is the use of NAD+ as a cofactor by the sirtuin class of histone deacetylases. The latter enzymes often function as NAD+/NADH sensors [95], which may also explain the marked upregulation of acetyl-lysine under conditions of altered NAD+/NADH ratio observed in hematologic malignancies such as MDS [31].

Methylation is also affected by the intracellular levels of metabolites; specifically, the methylation potential of the cell is determined by the ratio of SAM: S-adenosylhomocysteine (SAH). Methylation reactions normally proceed only if the levels of SAM are in sufficient excess over SAH, while the accumulation of SAH inhibits methylation [96]. In different cultured mixed lineage leukemia cell lines and patient blasts, as well as in BCR-ABL driven K562 cells, the disruption of SAM metabolism either pharmacologically or through nutritional deprivation in the culture media led to a reduction in global H3K79me2 levels [97]. This modification is catalyzed by disruptor of telomere silencing 1-like (DOT1L) and has been shown to be critical in leukemias bearing rearrangements of the gene encoding KMT2A for survival and maintenance of malignant potential. Interestingly, SAM deprivation led to a promoter-specific increase in H3K27me3 [97]. The explanation for the residue specific effects of SAM availability is not completely understood; however, it is possibly attributed to differential sensitivities of histone methyltransferases to its intracellular levels. Besides histone methyltransferases, SAM is also used by DNA methyltransferases; therefore, its levels have an impact on DNA methylation, which is also essential for accurate regulation of gene activity and other DNA-templated processes. The relevance of proper DNA methylation to hematologic malignancy is illustrated by the fact that about one out of three AML patients, mostly those with normal karyotype, carry mutations in the gene encoding the de novo DNA methyltransferase 3A (DNMT3A) [98,99]. These mutations are classified among the earliest events in leukemogenesis [100]. The modulation of SAM availability was also proposed to mediate the effect of the overexpression of a wild type metabolic enzyme, namely SHMT2, in driving oncogenesis in B-cell lymphoma 2 (BCL2)-expressing lymphomas. Overexpression of SHMT2, which is implicated in serine catabolism, led to epigenetic repression of the tumor suppressor genes encoding SAM and SH3 domain containing 1 (*SASH1*) and protein tyrosine phosphatase receptor type M (*PTPRM*), driving lymphomagenesis [101].

Besides SAM, other metabolites are also utilized by certain chromatin modifying enzymes. For instance, the Jumonji C (JmjC) family demethylases as well as the DNA oxygenases ten-eleven-translocation 1-3 (TET1-3) belong to the superfamily of 2-Oxoglutarate-dependent dioxygenases (2OGDDs), which use α-KG (also known as 2OG), Fe(II), and molecular oxygen for their catalytic functions, while they produce a hydroxylated product, succinate and carbon dioxide [20]. The catalytic reaction starts with the binding of the methyl-lysine substrate, α-KG and molecular oxygen to the enzyme’s active site. One of the two oxygen atoms reacts with the C–H bond of the methyl group and the other oxidizes α-KG to succinate and carbon dioxide. Following release from the catalytic site, the hydroxylated product can undergo non-enzymatic demethylation. 2OGDDs can function as oxygen sensors; however, their affinity for oxygen, iron and 2OG can vary among specific enzymes, suggesting that their activity can be differentially affected by the availability of the aforementioned metabolites. For instance, the KDM5A H3K4me3 and H3K4me2 demethylase is highly oxygen-dependent, and its acute inactivation phenocopies the effects of acute hypoxia, leading to increased H3K4 methylation. Similarly, the H3K27me3 demethylase KDM6A functions as an oxygen sensor and regulates cellular differentiation [102]. In T-ALL, KDM6A has a tumor suppressor role, with many patients carrying somatic loss-of-function mutations in the JmjC domain of the protein [103,104]. In some cases, though, the role of KDM6A in leukemogenesis is mediated through pathways independent of its demethylase activity [105]. On the other hand, the KDM3A demethylase, with preferential activity towards H3K9me2, has a lower O_2_ *K*_m_ value, allowing it to retain its catalytic function even under severe hypoxia [106]. Correspondingly, the activity of specific 2OGDDs can be differentially affected by iron levels, with enzymes that require high iron concentration for catalysis, such as KDM6B, TET1 and TET2, being more likely to be affected by iron deficiency [107]. Catalysis is also modulated by the levels of other factors that prevent iron oxidation, such as ascorbate, glutathione and cysteine, as well as by the redox state.

The sensitivities of specific 2OGDDs to oncometabolites, such as 2-HG, also vary, thereby the levels of these oncometabolites as well as the presence of IDH mutations can differentially affect the activity of the enzymes and, consequently, the levels of the specific methylation marks they modify [108]. The sensitivity of TET enzymes to 2-HG may provide an explanation of the aberrant DNA hypermethylation observed in IDH1/2 mutant cells in comparison to normal bone marrow. This notion is also supported by the fact that IDH1/2 and TET2 mutant AML cells display overlapping hypermethylation signatures, enriched for genes involved in pathways known to contribute to the malignant transformation of hematopoietic cells, such as genes that are direct targets of the GATA1 and GATA2 transcription factors [38].

Outside the 2OGDD family, another histone demethylase with relevance to hematologic malignancies, KDM1A (LSD1), uses FAD as a cofactor (Table 1). Besides FAD, the activity of LSD1 is also dependent on O_2_ availability, as O_2_ is required for the reoxidation of FADH_2_ back to FAD after a round of catalysis. Thus, under prolonged hypoxia, cellular FAD levels are gradually decreased. LSD1 catalyzes the demethylation of H3K4me2/1 and H3K9me2/1 and plays important roles both in normal hematopoiesis and in hematologic malignancy [109,110]. Upregulated levels of the protein are found in bone marrow biopsies of about one third of chronic myeloid leukemia (CML) patients and more than half of MDS patients. Furthermore, high levels of LSD1 expression are essential for MLL-R-induced leukemia, while the growth of AML cell lines is particularly sensitive to LSD1 depletion [111].

It should be noted that besides epigenetic enzymes that are directly regulated by oxygen, others are indirectly affected by oxygen availability, as they are transcriptional targets of HIF [106,112].

## 6. Concluding Remarks

**Figure 4:**
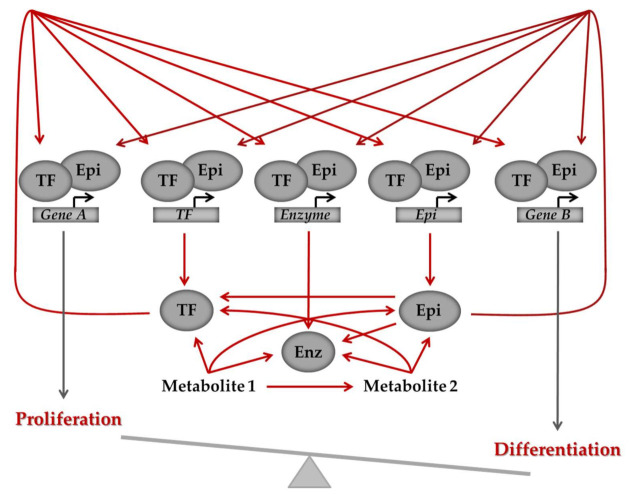
General model of the intricate interrelationships between the genome, metabolism and the epigenome. Defective regulation of the pathways can shift the balance between differentiation and proliferation and ultimately cause a transition from normal to malignant state. Arrows depict regulation, which can be activation or inhibition. Cross-talk between genes and metabolites is bi-directional, as illustrated by the arrows, with genes regulating metabolites, which, in turn, via their effect on epigenetic modifiers and transcription factors, signal back to the epigenome and regulate gene expression. Enz: metabolic enzyme, Epi: epigenetic regulator, TF: transcription factor.

The above examples provide a general overview of the intricate interrelationship between the genome (and epigenome) and metabolism in the context of hematologic malignancies (Figure 4). Genetic alterations that cause metabolic changes are among the driver mutations in blood neoplasms. In general, metabolic profiles adjusted to rapid energy production with a parallel increased synthesis of the key biomolecules to support growth and proliferation, and at the same time tailored to withstand the associated stress that results from the high metabolic rates, are thought to be favored in the course of malignant transformation. In parallel, metabolites can modulate the function of epigenetic enzymes and cause modifications of chromatin that can further induce transcriptional changes, eventually shifting the balance from differentiation towards proliferation. The dependence of several epigenetic modifiers on metabolites makes them promising therapeutic targets, as their activity and downstream effects can be modulated pharmacologically.

**Figure 5:**
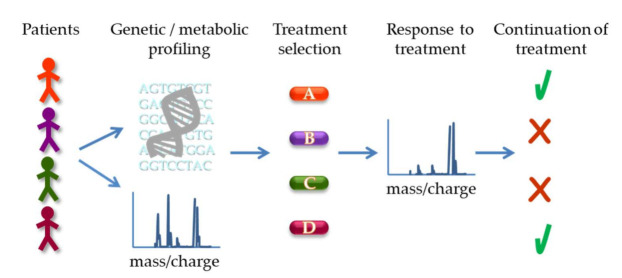
Potential application of genetic and metabolic profiling for treatment decision and response assessment. Patient samples can be subject to genetic sequencing to identify specific mutations as well as metabolomics analysis, e.g., by mass spectrometry, to profile metabolites (based on their mass/charge ratio schematically depicted). The results can guide the selection of the optimal treatment for the patient. Subsequently, metabolic analysis can also be used to assess response to therapy (illustrated as a shift in peaks in the spectrum) and determine continuation of the specific treatment or switch to a different approach.

Besides providing mechanistic insight into the underlying pathogenetic mechanisms, the development of high-throughput technologies for the analysis of the genome, epigenome, transcriptome and metabolome has a vast potential for translational application. In the context of hematologic malignancy, this potential is further increased by the use of single cell assays, which can unmask tumor heterogeneity and allow the identification of specific cell subpopulations of clinical importance (Figure 5). Next-generation sequencing technologies allow for the detection of genetic mutations in patient samples and the identified mutations can be informative on the aggressiveness of the tumor, as well as predict its response to specific therapies. Moreover, metabolic characterization of patient samples, based on mass-spectrometric and other technologies, can also be of use. Panels of metabolites can serve as biomarkers to define signatures that would predict and discriminate sensitivity from resistance and provide a means for the early assessment of response to therapy. Integrated analysis of the genetic, epigenetic and metabolic profile of the tumor may offer optimized solutions for the treatment of hematologic malignancies.

Despite the complexity of metabolic changes in cancer, the understanding of the key mediators of their effects offers significant potential for therapeutic intervention; the targeting of specific metabolic pathways and epigenetic modifiers, either individually or in combination, is a promising approach in the treatment of hematologic malignancy.

## Figures and Tables

**Table 1:** Examples of epigenetic modifying enzymes whose activity is affected by specific metabolites.

Metabolite	Factor	Activity
Acetyl-CoA	Histone acetyltransferases	Histone acetyltransferases
α-KG	KDM2B, KDM3A, KDM5A, KDM6A, KDM6B	Histone demethylases
α-KG	TET1-3	DNA oxygenases
FAD	KDM1A	Histone demethylase
Fe(II)	KDM2B, KDM3A, KDM5A, KDM6A, KDM6B	Histone demethylases
Fe(II)	TET1-3	DNA oxygenases
NAD+	Sirtuins	Histone deacetylases
Oxygen	KDM2B, KDM3A, KDM5A, KDM6A, KDM6B	Histone demethylases
Oxygen	TET1-3	DNA oxygenases
Oxygen	KDM1A	Histone demethylase
SAM	DNMT1, DNMT3A	DNA methyltransferases
SAM	KMT2A, NSD2	Histone methyltransferases

Acetyl-CoA: acetyl-coenzyme A, α-KG: α-ketoglutarate, FAD: flavin adenine dinucleotide, NAD+: nicotinamide adenine dinucleotide, SAM: S-adenosylmethionine.
